# Building an Effective International Medical Evacuation Program for Ukrainian Patients With Cancer Amid Prolonged Military Conflict

**DOI:** 10.1200/GO-24-00363

**Published:** 2024-10-10

**Authors:** Inesa Huivaniuk, Viacheslav Kopetskyi, Taras Ivanykovych, Andrei Nikiforchin, Darya Kizub, Marta Antoniv, Ali Dzhemiliev, Brittany Powell, Saar Yaniuta, Arman Kacharian, Anna Podolianko, Nelya Melnitchouk

**Affiliations:** ^1^ Department of Liver, Pancreatic and Peritoneal Carcinomatosis Surgery, Kyiv Regional Cancer Center, Kyiv, Ukraine; ^2^ Global Medical Knowledge Alliance, Boston, MA; ^3^ Danylo Halytsky Lviv National Medical University, Lviv, Ukraine; ^4^ Breast Medical Oncology, Division of Cancer Medicine, University of Texas MD Anderson Cancer Center, Houston, TX; ^5^ Division of General and GI Surgery, Department of Surgery, Brigham and Women's Hospital, Harvard Medical School, Boston, MA; ^6^ Center for Surgery and Public Health, Brigham and Women's Hospital, Harvard Medical School, Boston, MA; ^7^ Ministry of Health of Ukraine, Kyiv, Ukraine; ^8^ Medical Evacuation Coordination Unit, Ministry of Health of Ukraine, Kyiv, Ukraine

## Abstract

**PURPOSE:**

During military conflicts, the immediate response to a severely disrupted health care system often overlooks the needs of patients with cancer who require continuous specialized care. The full-scale Russian invasion of Ukraine in February 2022 was no exception, leaving many Ukrainian patients without access to essential care.

**MATERIALS AND METHODS:**

We conducted a retrospective cohort study to assess the impact of the MedEvac program, facilitating the transfer of Ukrainian patients with cancer to European Union (EU) institutions for treatment, and to describe its components. Patient data from the Ministry of Health of Ukraine (MOH) database (April 2022-April 2023) were analyzed.

**RESULTS:**

Of 639 applications in the MOH database, 339 (53.1%) had sufficient data for analysis and, of those, 281 (82.9%) were evacuated to EU hospitals. Median age of evacuated patients was 47 (IQR, 38-58) years and most were newly diagnosed (94.0%, n = 264). Predominantly, patients were evacuated for systemic cancer therapy (81.9%, n = 230). Multivariate logistic regression analysis revealed that a good performance status (Eastern Cooperative Oncology Group 0-2) was the most significant factor associated with evacuation (odds ratio [OR], 9.64 [95% CI, 3.08 to 30.23]). Patients with melanoma were more likely to be evacuated, even after adjustment for performance status (OR, 2.56 [95% CI, 1.14 to 5.72]), while patients with head and neck cancer were significantly less so (OR, 0.20 [95% CI, 0.06 to 0.72]).

**CONCLUSION:**

MedEvac program provides a viable model for medical evacuation and management of patients with cancer amid prolonged military conflict, highlighting the importance of international cooperation and setting a precedent for other crisis responses. Continuous evaluation and adaptation are essential to ensure the program's effectiveness and sustainability.

## Teaser

Explore the first detailed report @JCOGO_ASCO on international #MedEvac program, evacuating #cancer patients amid #war ❤.

## INTRODUCTION

The impact of armed conflicts on a health care system is always profound and multifaceted—damage to medical facilities and equipment, internal and external migration of personnel, and disruption of health care logistics and supply chains to name just a few.^1^ Although the immediate consequences of any crisis, such as casualties, injuries, and mass displacement, capture global attention, the disastrous effect on patients with cancer typically remains in the shadows.^2^ This vulnerable population requires specialized, multicomponent, and continuous care, which cannot be provided adequately in circumstances of a destroyed health care framework.^2,3^ This poses a significant challenge for cancer specialists, forcing them to rapidly reorganize and adapt care delivery within a war-torn country and turn to neighboring nations for assistance.^4,5^

**CONTEXT**

**Key Objective**
What does it take to establish an efficient medical evacuation program for patients with cancer amid prolonged military conflict?
**Knowledge Generated**
The first detailed analysis, to our knowledge, of the MedEvac program for patients with cancer from war-affected regions of Ukraine highlights its structure, evacuation steps, and intermediate outcomes. In the first year, 281 adult patients were transferred to European Union institutions, primarily for systemic cancer therapy (81.9%, n = 230). Eastern Cooperative Oncology Group 0-2 performance status was the most significant factor associated with medical evacuation.
**Relevance**
The MedEvac program shows a viable model for maintaining cancer care during conflicts, emphasizing the importance of continuous international support, clear eligibility criteria, and ongoing adaption to evolving needs.

The full-scale Russian invasion of Ukraine in February 2022 immediately left thousands of Ukrainian patients with cancer unable to see their doctors, obtain essential medications, or access specialized services.^6-11^ A strategic impact assessment early in the conflict estimated that 60%-70% of cancer care in Ukraine had been disrupted in only the first month of the invasion, with around 33,000 patients with cancer among the refugee population expected over the following year.^12^ In response, the European Union (EU) and Ukraine launched the MedEvac program—a collaborative initiative addressing medical evacuation and treatment of Ukrainian patients with cancer abroad. Drawing on insights from previous experiences in Iraq and Syria, which highlighted the demand for systematic health care solutions during protracted military conflicts, this program represents a pioneering effort to create a long-term solution for medical evacuation and cancer care at the level of health care systems.^2,13,14^ It also underscores the critical need for international cooperation in times of crisis and sets a replicable precedent for future responses to similar dire events.

## MATERIALS AND METHODS

### Study Design, Data Source, and Setting

We described the MedEvac program's structure and outlined key steps of the evacuation of patients with cancer from Ukraine to EU institutions on the basis of data provided by program implementers, who include the authors of this paper. A retrospective cohort study was conducted to analyze the intermediate outcomes of the MedEvac program and explore the factors associated with patient evacuation. Data were retrieved from the Ministry of Health of Ukraine (MOH) database (April 2022-April 2023), which contains applications for participation in the MedEvac program. As part of the application process, patients consented to the use of their deidentified data for research purposes. The database's creation and maintenance followed the directives outlined in the MOH Order for validation of criteria for transferring Ukrainian citizens for treatment abroad and the list of medical institutions, which coordinate transferring Ukrainian citizens for treatment abroad during wartime.^15^ The database has been maintained by a team of 11 coordinators at MOH and funded by the European Commission.

### Statistical Analysis

Patient characteristics were compared between the evacuated and nonevacuated cohorts using the Mann-Whitney *U* test for continuous variables and the chi-square test or Fisher's exact test for categorical variables, as appropriate. Statistical significance was two-sided and set as *P* < .05. Given the significant variety of cancer diagnoses in our study cohort, logistic regression analysis was performed separately for each diagnosis and for other factors to determine their association with medical evacuation. Initially, the univariate analysis was conducted to identify potential factors, and variables with *P* < .20 were then included in the explanatory multivariate model to describe factors independently associated with patient evacuation. The results of the logistic regression analysis are presented as odds ratios (ORs) with 95% CI. Statistical analyses were performed using IBM SPSS Statistics software (version 23.0; IBM Corporation; Armonk, NY).

### Institutional Review Board Approval and Ethical Considerations

This study involved the analysis of preexisting deidentified patient data retrieved from the MOH database. It did not meet the criteria for research involving human subjects; therefore, institutional review board approval was not required.

## RESULTS

### The MedEvac Program

In early March 2022, MOH sent an open letter to the health care division of the EU, requesting urgent assistance for patients with cancer amid the escalating medical crisis triggered by the full-scale Russian invasion of Ukraine. The program garnered participation from all EU nations, categorized as Union Civil Protection Mechanism (UCPM) member states, along with eight additional countries willing to contribute as UCPM participating states (Data Supplement, Fig S1).^16^ Given the extensive number of participating countries, the multiple steps involved in the evacuation process, and the complex coordination required, the program necessitated the involvement of several supranational organizations, including the Emergency Response Coordination Centre (ERCC) and the WHO.^17^ Initially, Ukrainian cancer centers had the autonomy to select patients for evacuation. However, to enhance efficiency and ensure fairness as the program expanded, a panel of experts from MOH and ERCC established robust patient eligibility criteria (Table 1) and developed a systematic evacuation algorithm (Table 2). The MedEvac program has focused exclusively on adult patients with cancer, while Ukrainian children diagnosed with solid and hematologic malignancies and requiring immediate treatment have been evacuated abroad through another international collaboration—Supporting Action for Emergency Response in Ukraine (SAFER Ukraine), developed in parallel.^18,19^

**TABLE 1:** Medical Evacuation Eligibility Criteria

Requirement
Age requirement: must be 18 years or older
Cancer diagnosis: must have a verified diagnosis and stage of malignancy
Satisfactory performance status
ECOG score between 0 and 2
Karnofsky Performance Status from 60 to 100, indicating medical stability for travel
Treatment with a curative intent
Chemotherapy (if specific necessary drugs are unavailable locally)
Target therapy
Immunotherapy
Hematopoietic stem-cell transplantation
Stereotactic radiotherapy
Brachytherapy
Complex surgical procedures with advanced reconstruction
Legal documentation: must possess an international ID; additionally, men must provide a border-crossing permit, such as a disability certificate or a military ID that includes a notation of unfitness for military service
Communication: ability to communicate with English-speaking personnel independently or through a caregiver

Abbreviations: ECOG, Eastern Cooperative Oncology Group; ID, identification.

**TABLE 2:** Algorithm of Patient Evacuation

Step	Description	Roles and Responsibilities
1. Application submission	Patients or their representatives submit applications to MOH through one of the channels. They include direct submissions via the MOH website, submissions through oncologists, who coordinate with regional Health Departments, or through Ukrainian NGOs working with patients with cancer	Oncologists and NGOs: identification of eligible patients and assistance with application submission Patients: application submission. MOH: application reception and website maintenance
2. Application review	A designated group within MOH reviews applications and assesses patients' eligibility. Approved applications are forwarded to ERCC via e-mail or EWRS as evacuation requests. The ERCC team adds evacuation requests to the patient database, notifies health care authorities of UCPM MS/PS (Data Supplement, Fig S1), and facilitates communication between them, MOH, and the MedEvac Hub	MOH: application review, translation of medical records to English, and evacuation request submission EWRS: facilitation of communication between MOH and ERCC ERCC: evacuation request processing and communication with health care authorities of UCPM MS/PS, MOH, and MedEvac Hub CECIS and EWRS: patient database management
3. Patient selection	UCPM reviews evacuation requests in the database within 5-7 days and suggests appropriate EU institutions for treatment. The ERCC team communicates these offers to MOH, which reviews them and informs the patient	UCPM: review and selection of evacuation requests ERCC: communication with UCPM MS/PS and MOH MOH: review of EU institution offers and communication with patients
4. Evacuation preparation	After patient selection, ERCC starts planning their evacuation through the MedEvac Hub. Patients secure necessary documentation for crossing the Ukraine-Poland border, such as an international ID and, for men, a border-crossing permit (a disability certificate or a military ID with a note of unfitness for military service)	ERCC: coordination of patients' evacuation logistics Patients: preparation of documents for crossing the Ukraine-Poland border
5. Arrival at the MedEvac Hub	Patients arrive in Lviv, Ukraine, cross the border, and arrive at MedEvac Hub in Jasionka near Rzeszów, Poland (Data Supplement, Fig S1). The MedEvac Hub ensures preflight medical stability and provides 24/7 nurse care, including communicable disease screenings, vaccinations, and mental health support	GPMG and NGOs: financing transportation to Lviv WHO: EMS transportation funding from Lviv to Jasionka DG ECHO and PCPM: MedEvac Hub operation and medical service provision
6. Departure from the MedEvac Hub	Patients depart from the MedEvac Hub via weekly flights on a medical plane and then arrive at the host EU institution. Caregivers can accompany patients on board and at the MedEvac Hub; however, their postarrival accommodation is not guaranteed	ERCC, UCPM MS/PS, and EU: transportation financing
7. Treatment at the EU institution	Patients receive necessary medical treatment at EU institutions, facilitated by the activation of temporary protection status by the ERCC. This ensures that patients receive care in accordance with EU standards	ERCC: temporary protection status activation Health care authorities of UCPM MS/PS: treatment funding
8. Return to Ukraine	After treatment, patients return to Ukraine for continued rehabilitation and monitoring	Patients (occasionally UCPM MS/PS): financial coverage of return to Ukraine, including repatriation of mortal remains

Abbreviations: CECIS, Common Emergency Communication and Information System; DG ECHO, Directorate-General for European Civil Protection and Humanitarian Aid Operations; EMS, Emergency Medical Services; ERCC, Emergency Response Coordination Centre; EU, the European Union; EWRS, Early Warning and Response System; GPMG, Governmental Program of Medical Guaranties; ID, identification; MOH, the Ministry of Health of Ukraine; MS/PS, member states/participating states; NGO, nongovernmental organization; PCPM, Polish Center for International Aid; UCPM, Union Civil Protection Mechanism.

### Study Population

**FIG 1:**
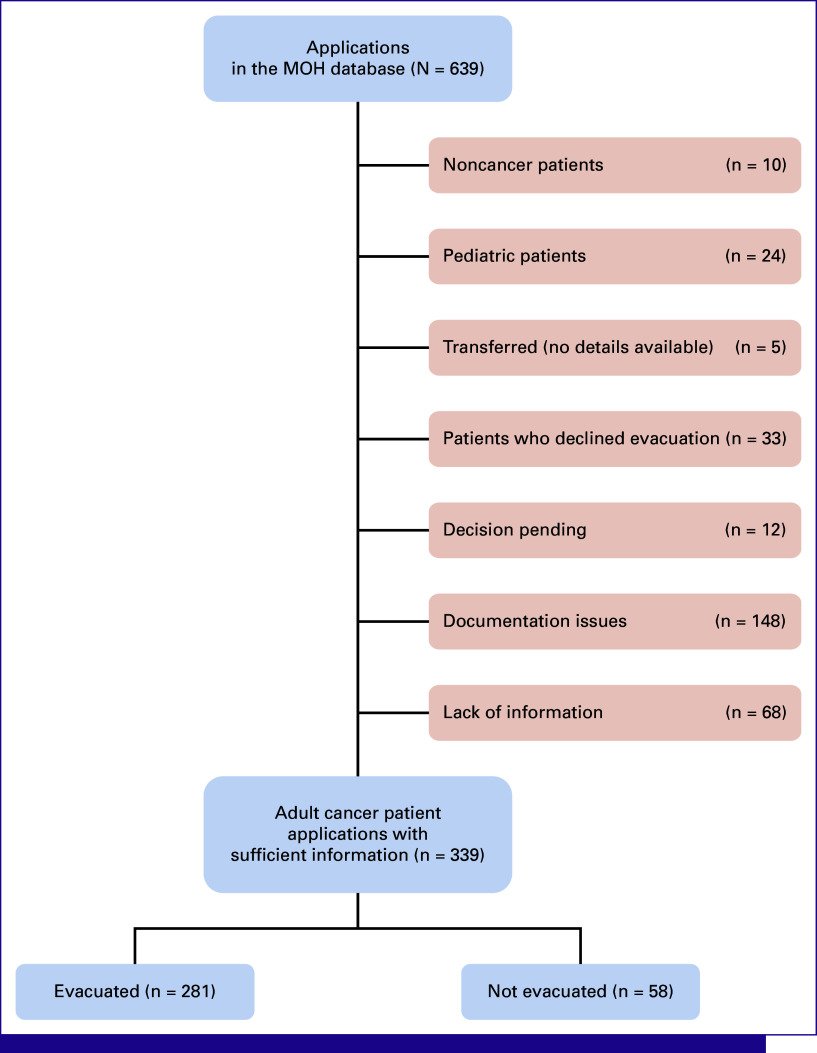
Study flowchart. MOH, Ministry of Health of Ukraine.

**FIG 2:**
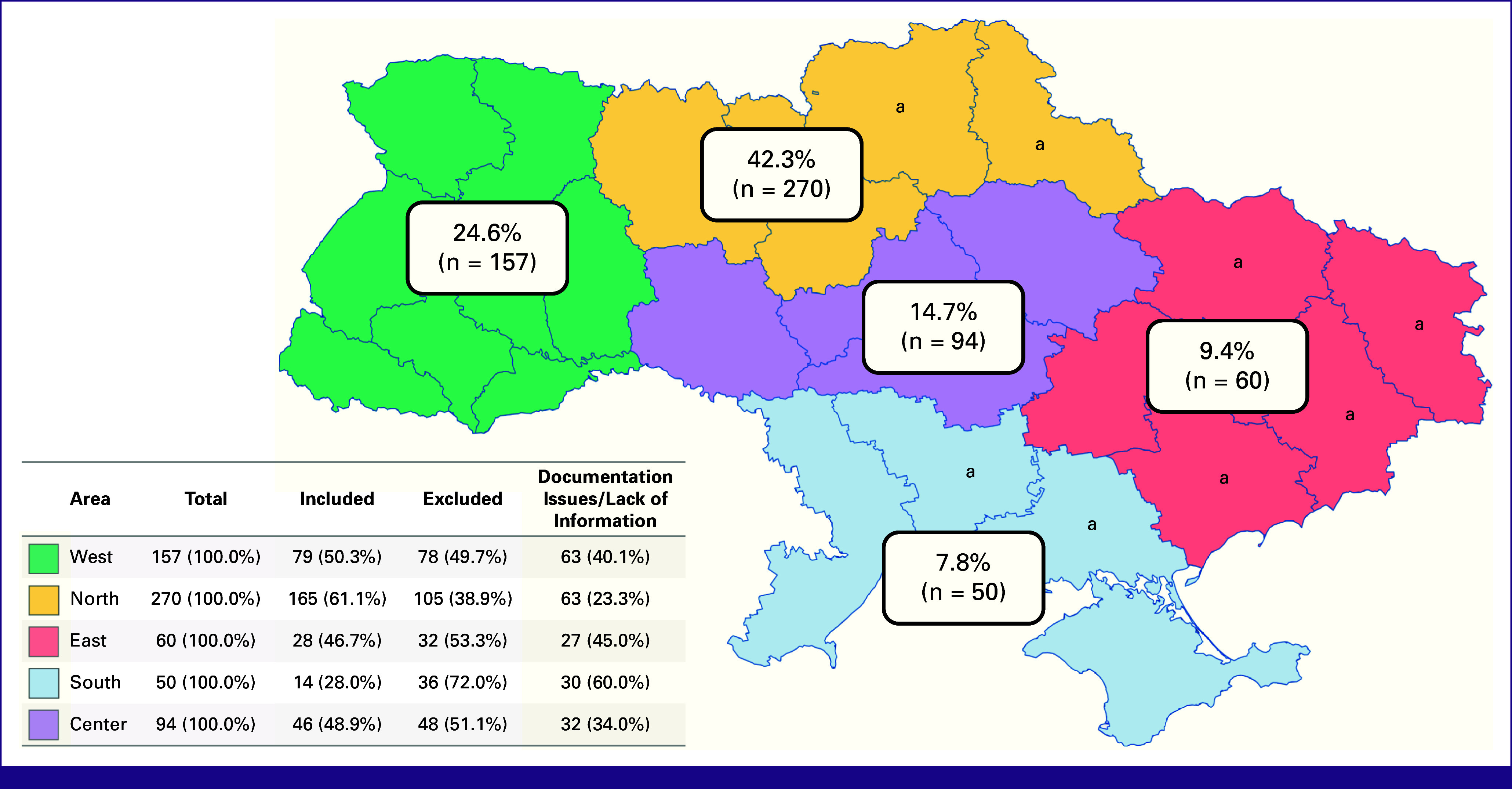
Geographical distribution of applications in the MOH database. MOH, the Ministry of Health of Ukraine. ^a^Regions of most active combat in April 2022-April 2023.

A total of 639 applications were identified in the MOH database. Of these, 300 (46.9%) were excluded because of various reasons, with medical documentation issues (n = 148, 23.2%) and lack of information (n = 68, 10.6%) being predominant (Fig 1). However, nearly all applications (n = 631, 98.7%) in the database had a region code, enabling the construction of an application distribution map (Fig 2). The lowest numbers of applications were from the East (n = 60, 9.4%) and the South (n = 50, 7.8%) of Ukraine, with significant proportions excluded also because of documentation issues and incomplete information. Overall, 339 (53.1%) adult cancer patient applications with sufficient data were included in this study. Among these, 281 (82.9%) patients were evacuated to EU institutions and 58 (17.1%) were not.

### Patient Characteristics

**FIG 3:**
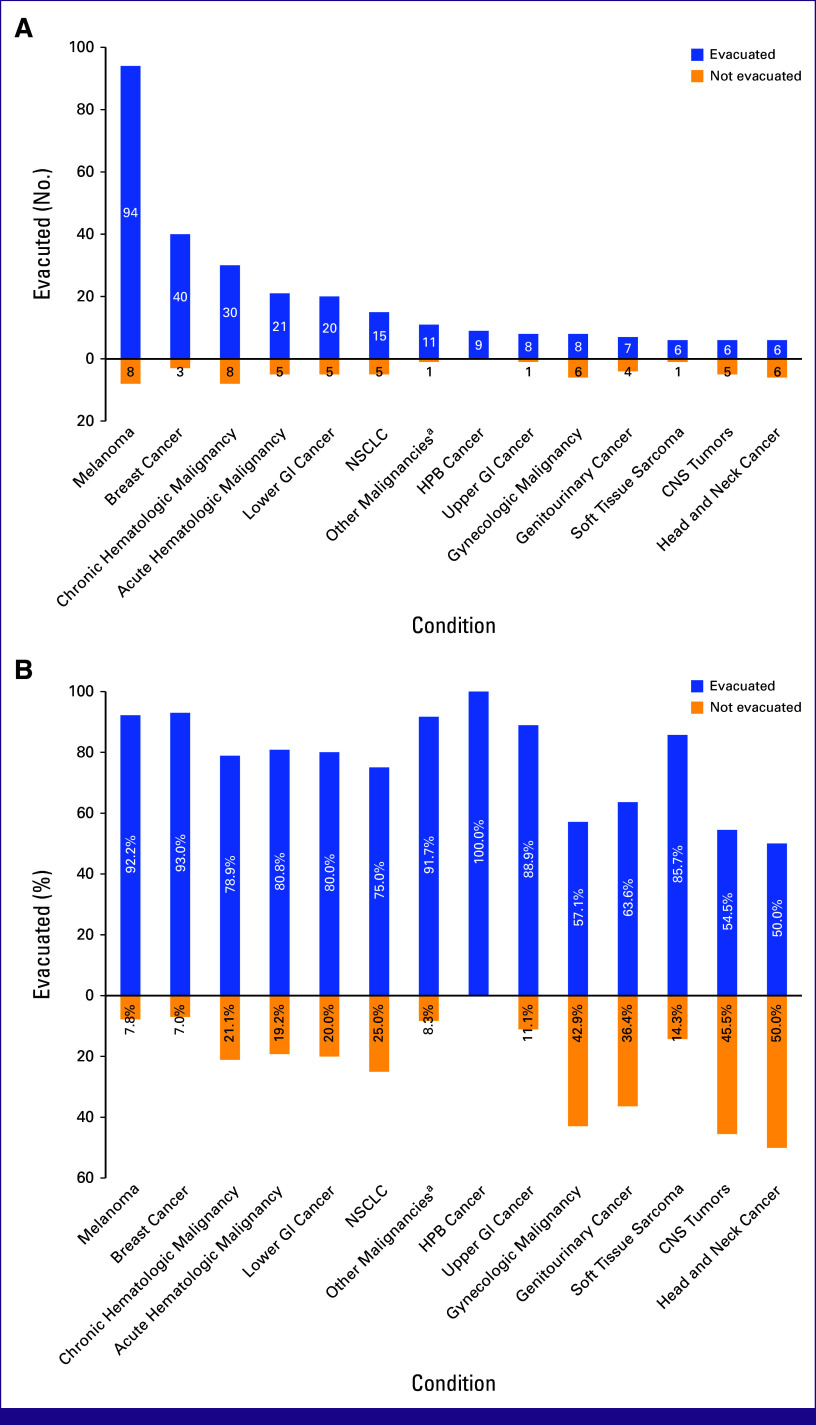
Patient distribution by diagnosis in (A) absolute numbers and (B) percentages. HPB, hepatopancreatobiliary; NSCLC, non–small cell lung cancer. ^a^Other malignancies include skin cancer, mesothelioma, and endocrine tumors.

The median age for the evacuated cohort was 47 (IQR, 38-58) years, while for the nonevacuated cohort, it was 45 (IQR, 36-57) years (*P* = .36). Patient distribution by diagnosis in absolute numbers and percentages is detailed in Figure 3. The most common diagnoses were melanoma (33.5% [n = 94] of evacuated *v* 13.8% [n = 8] of nonevacuated cohort; *P* < .01), breast cancer (14.2% [n = 40] of evacuated *v* 5.2% [n = 3] of nonevacuated cohort; *P* = .06), and chronic hematologic malignancy (10.7% [n = 30] of evacuated *v* 13.8% [n = 8] of nonevacuated cohort; *P* = .49). The proportions of patients with disease recurrence did not differ between the cohorts: 17 (6.0%) and 4 (6.9%), *P* = .77, respectively. Comorbidities were present in 26.0% (n = 73) of the evacuated and 36.2% (n = 21) of the nonevacuated patients, yet this difference was not significant (*P* = .11). In turn, Eastern Cooperative Oncology Group (ECOG) performance status differed significantly, with a higher percentage of patients with ECOG 0-2 in the evacuated cohort (87.9% [n = 247] *v* 81.0% [n = 47]; *P* < .01). Systemic cancer therapy was the most commonly required treatment in both cohorts—in 81.9% (n = 230) and 82.8% (n = 48) of patients, respectively (*P* = .73; Table 3).

**TABLE 3:** Patient Characteristics

Variable	Evacuated (n = 281)	Not Evacuated (n = 58)	P
Age, years, median (IQR)	47 (38-58)	45 (36-57)	.36
Age ≥65 years, No. (%)	26 (9.3)	6 (10.3)	.80
Disease status, No. (%)			
Newly diagnosed	264 (94.0)	54 (93.1)	.77
Recurrence	17 (6.0)	4 (6.9)	
Comorbidities, No. (%)	73 (26.0)	21 (36.2)	.11
Performance status, No. (%)			
ECOG 0-2	247 (87.9)	47 (81.0)	**<.01**
ECOG 3-4	5 (1.8)	9 (15.5)	
Unknown	29 (10.3)	2 (3.4)	
Required treatment, No. (%)			
Systemic therapy	230 (81.9)	48 (82.8)	
Surgery	11 (3.9)	2 (3.4)	.73
Radiation therapy	13 (4.6)	1 (1.7)	
HCT or OT	27 (9.6)	7 (12.1)	
Rationale for no evacuation, No. (%)			
MOH rejection		28 (48.3)	
Patient refusal	NA	9 (15.5)	NA
Decision is pending		21 (36.2)	

NOTE. The *P* value in bold denotes statistical significance.

Abbreviations: ECOG, Eastern Cooperative Oncology Group; HCT, hematopoietic cell transplantation; MOH, the Ministry of Health of Ukraine; NA, not applicable; OT, organ transplantation.

### Logistic Regression Analysis

Univariate and multivariate logistic regression analyses identified that a good performance status (ECOG 0-2) was significantly associated with a higher likelihood of medical evacuation (OR, 9.64 [95% CI, 3.08 to 30.23]; *P* < .01). Patient age, presence of comorbidities, disease status, and required treatment were not significant factors. Among various cancer diagnoses, melanoma was associated with a higher likelihood of patient evacuation, both before (OR, 3.14 [95% CI, 1.43 to 6.90]; *P* < .01) and after adjustment for performance status (OR, 2.56 [95% CI, 1.14 to 5.72]; *P* = .02). Conversely, patients with head and neck cancer were significantly less likely to be evacuated: unadjusted OR, 0.19 (95% CI, 0.06 to 0.61; *P* < .01), and adjusted OR, 0.20 (95% CI, 0.06 to 0.72; *P* = .01; Data Supplement, Tables S1 and S2).

## DISCUSSION

To the best of our knowledge, this is the first study detailing the structure and assessing the intermediate outcomes of an international medical evacuation program for patients with cancer during wartime. Prolonged conflicts with continuous targeting of humanitarian and health care infrastructure, like the ongoing war in Ukraine, are known for significant disruption of specialized medical care, including oncology.^2,6,9-11,20-22^ Addressing this complex challenge requires a multifaceted strategy, including reorganizing and optimizing remaining resources, restoring destroyed facilities, and evacuating patients who temporarily cannot receive treatment within the country or abroad.^14^ Some studies from other conflict zones have emphasized the importance of robust external support to maintain adequate cancer care amid ongoing violence.^23,24^ International medical evacuation becomes one of the key components of this support, enabling patients to continue cancer therapy and access treatments unavailable in their home country.^8,10,11,14^ Given that many military conflicts are protracted and characterized by fluctuating levels of violence and destruction, evacuation programs must be long-lasting and adaptable to meet the needs of the most vulnerable patients.

Building such a program involves several critical elements. First, there must be efficient communication between MOH, regional health departments, primary oncologists, and nongovernmental organizations (NGOs) working with patients with cancer. This network is vital for referring patients with cancer to specialists, facilitating the application process, and raising awareness about the functioning evacuation program, especially in areas with active combat and significant deprivation from specialized cancer care (Fig 2).^8^ The MOH leadership in this framework also allows for independent assessment of medical documents and their translation, acquisition of patient data, and funding for transfers within Ukraine. Additionally, MOH can secure border-crossing permits for male patients during martial law and manage official communication with health care authorities of other countries. Collaboration with supranational organizations, such as UCPM, ERCC, and WHO, is crucial as it provides access to extensive international health care and logistical networks (Table 2). This cooperation can also ensure stable health care and transfer financial coverage, critical for the sustainability of a program of this scale,^13,25^ which in the case of MedEvac, was supported by the EU temporary protection mechanism, guaranteeing Ukrainian patients free access to medical care across all EU states.^26^ Intermediate transportation hubs with 24/7 medical care, organized by national medical authorities and NGOs, play a significant role in evacuation process safety. Additionally, telemedicine can facilitate international communication between patient's primary and foreign medical teams and can also be used for post-treatment follow-up.^6,14,27,28^

The MedEvac program has demonstrated viability and continues to operate despite ongoing challenges. The evacuated patients had a wide spectrum of cancer diagnoses, including hematologic malignancies and conditions requiring organ transplantation (Table 3; Fig 3). The ECOG score was the most important factor associated with patient evacuation, while age and comorbidities were not found to be significantly associated with it (Data Supplement, Table S1). Since the launch of MedEvac, some patients have begun to understate the severity of their condition to improve their chances of selection, causing issues upon arrival for treatment, when the host country's medical team did not consider them proper candidates for the planned treatment. To address this problem, we established strict patient eligibility criteria, involving an expert group from the ERCC, and delegated decision making to MOH experts (Table 1). This approach helped mitigate the problem and made the patient-selection process more effective and fair, with 82.9% (n = 281) of analyzed patients with cancer evacuated and 36.2% (21/58) of the nonevacuated cohort awaiting a decision at the moment of data extraction.

Despite its accomplishments, the MedEvac program has areas for improvement. The lack of follow-up and treatment outcomes data limits a comprehensive assessment of the program's effectiveness in terms of patient survival and quality of life. We strongly advocate for incorporating continuous communication and data collection to track patient progress and outcomes in this program and future similar initiatives. Additionally, adapting the program to current needs is essential. Over time, there may be changes in diagnoses of patients needing evacuation and a shift in the most required treatment from systemic cancer therapy to radiotherapy because of escalating attacks on the Ukrainian electricity grid and specialized medical facilities along with simultaneous restoration of certain drug and medical supply chains.^6,22,29^ At the same time, the influx of patients requiring advanced treatment has strained drug stocks in EU countries. We see a potential solution in inviting other countries to participate in MedEvac, strategically planning and procuring medications by host countries on the basis of the anticipated modeled numbers of evacuees, and most importantly, in restoring Ukrainian national health care and pharmaceutical systems.^8,10,12,13,29,30^ Efficient resource utilization, such as using returning air and emergency medical services transportation for previous patients and donated supplies, can optimize costs and benefit both the MedEvac Hub in Jasionka, Poland, and Ukrainian institutions (Table 2). Finally, the quality of acquired data is another area of concern. Many incomplete applications were excluded from the analysis because of medical documentation issues (n = 148, 23.2%) and lack of information (n = 68, 10.6%; Fig 1). This problem was observed across all geographic areas, not only in the East and South (n = 27, 45.0%, and n = 30, 60.0%, respectively), directly affected by active combat, but also in the West (n = 63, 40.1%), where many citizens have moved since the beginning of the conflict, further straining the already overburdened health care system (Fig 2).^8,10,22^ To address the issue of insufficient data collection, it is critical to implement standardized, concise, yet informative application forms, such as the one proposed by our team (Data Supplement, Table S3).

This study has a few intrinsic limitations, including the retrospective nature of the data and challenges in its collection because of the ongoing armed conflict. Additionally, there were many patients with cancer, who left Ukraine independently, particularly during the initial months of the invasion, and their numbers are hard to estimate. The rapidly evolving nature of the conflict and health care situation in Ukraine also means that the data captured may quickly become outdated, necessitating continual reassessment of the current state of national cancer care and the program's effectiveness. Despite these limitations, this study provides critical insights into the implementation and intermediate outcomes of the MedEvac program, establishing a template for international cooperation in crises. By sharing our findings, we aim to inform and inspire similar efforts in other conflict-affected regions, ultimately improving access to essential cancer care for vulnerable populations worldwide.

In conclusion, the MedEvac program, a collaborative effort of the EU and Ukraine, has proven to be a vital response to the health care crisis caused by the Russian invasion. By successfully evacuating a significant number of Ukrainian patients with cancer with various diagnoses, the program demonstrated its feasibility and underscored the critical importance of international cooperation in building a long-term, viable system to address cancer care challenges in prolonged military conflicts. Systemic cancer therapy was the most common modality of required treatment. In terms of diagnosis, patients with melanoma constituted a significant portion of the evacuated population, while a good performance status (ECOG 0-2) was the most important factor associated with medical evacuation. This study also highlights the MedEvac program's key components, such as establishing robust patient eligibility criteria, developing a systematic evacuation algorithm, and ensuring effective coordination among multiple national and supranational organizations. Moving forward, the MedEvac model can serve as a blueprint for future initiatives aimed at meeting similar health care needs in current and future crises worldwide. Continuous evaluation and adaptation of such programs are essential to ensure their effectiveness, sustainability, and ability to meet evolving health care demands.

## Data Availability

The data set used and analyzed during the current study is available from the corresponding author upon reasonable request.
